# The genome sequence of a segmented worm, *Terebella lapidaria *Linnaeus, 1767

**DOI:** 10.12688/wellcomeopenres.22823.1

**Published:** 2024-08-07

**Authors:** Teresa Darbyshire, Patrick Adkins, Anna Holmes, John Bishop, Nova Mieszkowska, Marine Biological Association Genome Acquisition Lab, Darwin Tree of Life Barcoding collective, Wellcome Sanger Institute Tree of Life Management, Samples and Laboratory team, Wellcome Sanger Institute Scientific Operations: Sequencing Operations, Wellcome Sanger Institute Tree of Life Core Informatics team, Tree of Life Core Informatics collective, Darwin Tree of Life Consortium

**Affiliations:** 1 Amgueddfa Cymru, Cardiff, Wales, UK; 2 The Marine Biological Association, Plymouth, England, UK

**Keywords:** Terebella lapidaria, a segmented worm, genome sequence, chromosomal, Terebellida

## Abstract

We present a genome assembly from an individual *Terebella lapidaria* (segmented worm; Annelida; Polychaeta; Terebellida; Terebellidae). The genome sequence spans 765.20 megabases. Most of the assembly is scaffolded into 16 chromosomal pseudomolecules. The mitochondrial genome has also been assembled and is 15.97 kilobases in length.

## Species taxonomy

Eukaryota; Opisthokonta; Metazoa; Eumetazoa; Bilateria; Protostomia; Spiralia; Lophotrochozoa; Annelida; Polychaeta; Sedentaria; Canalipalpata; Terebellida; Terebelliformia; Terebellidae *; Terebella*; *Terebella lapidaria* Linnaeus, 1767 (NCBI:txid1131437).

## Background

*Terebella lapidaria* Linnaeus, 1767 is a large-bodied polychaete of the family Terebellidae sensu stricto, originally described from the Mediterranean Sea ( Gil, 2011; Lavesque *et al.*, 2021). It has also been recorded from the Adriatic and Aegean Seas as well as the Atlantic coast of France and the southern coast of the UK ( Fauvel, 1927; Gil, 2011; Lavesque *et al.*, 2021; Linnaeus, 1767; NBN Trust Partnership, 2024), although its status in Atlantic regions is considered uncertain at this time until specimens can be compared to those from the Mediterranean ( Lavesque *et al.*, 2021). Around the UK specifically, *Terebella lapidaria* has been recorded from Devon and Cornwall, along the south-west coast of England and the Bristol Channel, and is considered a non-native species of interest to Northern Ireland.

This species primarily inhabits shallow and intertidal waters, under rocks, in rock or shale crevices, shale gravel or muddy bottoms ( Gil, 2011; Lavesque *et al.*, 2021; Marine Biological Association, 1957). They can reach 80–160 segments in size and up to 9 centimetres in length ( Fauvel, 1927).

The genus *Terebella* is characterised by having notochaetae on more than 25 segments with no clear definition between thorax and abdomen, and three pairs of branched branchiae. Of 37 species currently recognised within the genus, only *T. lapidaria* and *Terebella banksyi* Lavesque, Daffe, Londoño-Mesa & Hutchings, 2021 occur either around or in close proximity to the UK. *Terebella banksyi* is currently known only from its type locality in Arcachon Bay, France. The two species can be distinguished through the placement of the branchial pairs (on segments II–IV on *T. lapidaria* and discontinuous on segments II–III and segment V on *T. banksyi*) and the number of nephridial and genital papillae (five pairs on *T. lapidaria*, twelve pairs on *T. banksyi*) ( Lavesque *et al.*, 2021).

The genome of *Terebella lapidaria* was sequenced as part of the Darwin Tree of Life Project, and represents the first of its kind for this species.

## Genome sequence report

**Figure 1.**
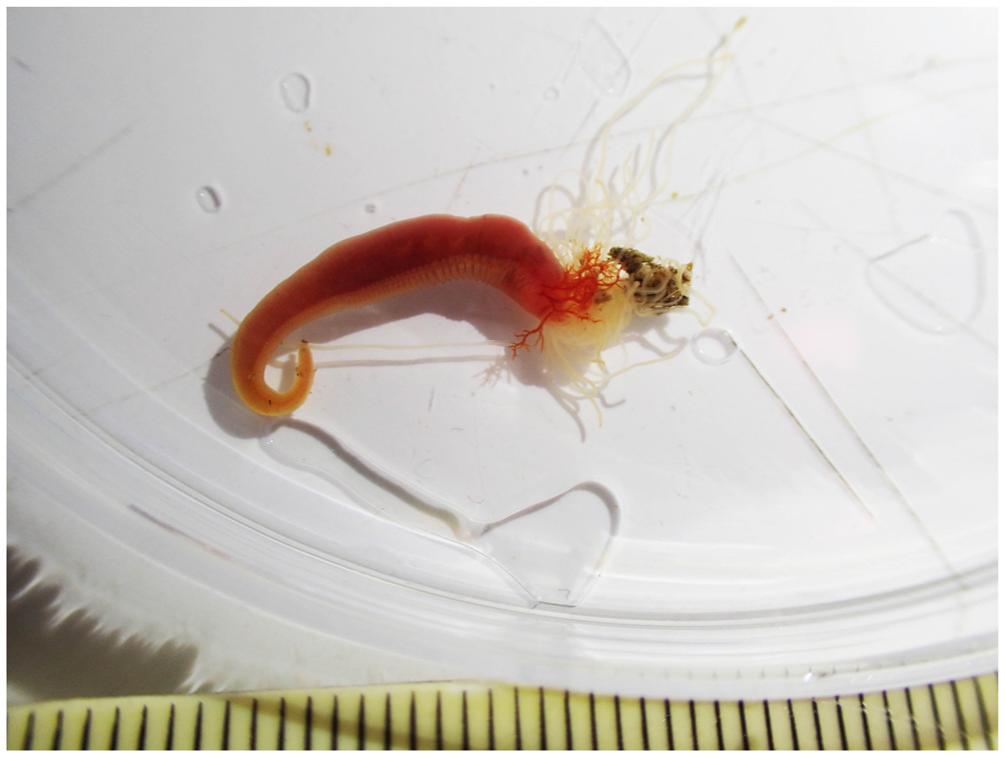
Photograph of the *Terebella lapidaria* (wtTerLapi1) specimen used for genome sequencing.

The genome of an adult *Terebella lapidaria* ( Figure 1) was sequenced using Pacific Biosciences single-molecule HiFi long reads, generating a total of 22.32 Gb (gigabases) from 2.37 million reads, providing approximately 34-fold coverage. Primary assembly contigs were scaffolded with chromosome conformation Hi-C data, which produced 137.71 Gbp from 911.98 million reads, yielding an approximate coverage of 180-fold. Specimen and sequencing information is summarised in Table 1.

**Table 1.** Specimen and sequencing data for *Terebella lapidaria*.

Project information			
**Study title**	Terebella lapidaria ((a segmented worm))		
**Umbrella BioProject**	PRJEB59382		
**Species**	*Terebella lapidaria*		
**BioSample**	SAMEA8724784		
**NCBI taxonomy ID**	1131437		
Specimen information			
**Technology**	**ToLID**	**BioSample accession**	**Organism part**
**PacBio long read sequencing**	wtTerLapi1	SAMEA8724848	Anterior body
**Hi-C sequencing**	wtTerLapi3	SAMEA110451062	Mid body
**RNA sequencing**	wtTerLapi3	SAMEA110451061	Posterior body
Sequencing information			
**Platform**	**Run accession**	**Read count**	**Base count (Gb)**
**Hi-C Illumina NovaSeq 6000**	ERR10851521	9.12e+08	137.71
**PacBio Sequel IIe**	ERR10841323	5.49e+05	4.02
**PacBio Sequel IIe**	ERR10841324	2.37e+06	22.32
**RNA Illumina NovaSeq X**	ERR12765137	6.82e+07	10.3

**Figure 2.**
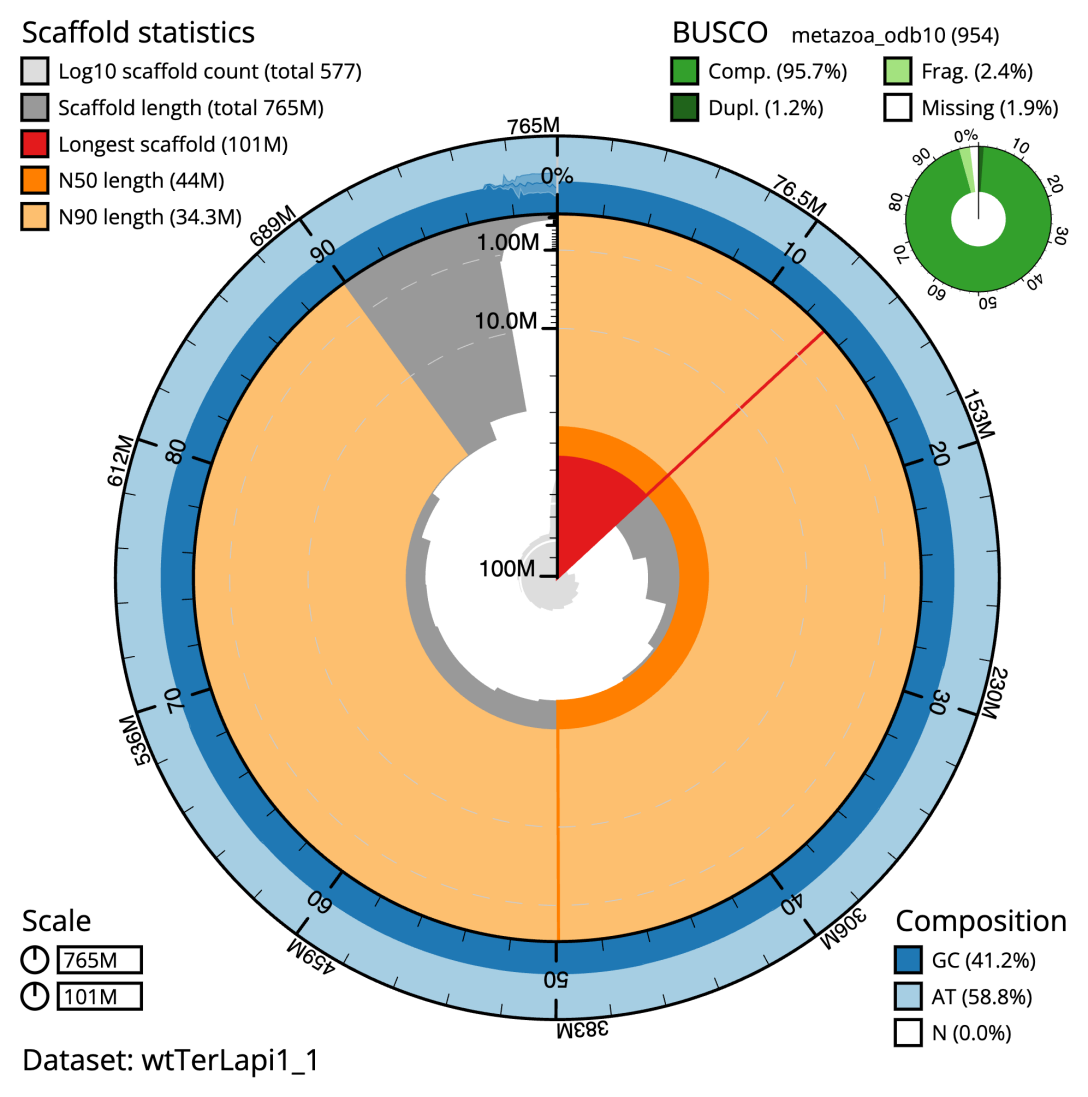
Genome assembly of *Terebella lapidaria*, wtTerLapi1.1: metrics. The BlobToolKit Snailplot shows N50 metrics and BUSCO gene completeness. The main plot is divided into 1,000 size-ordered bins around the circumference with each bin representing 0.1% of the 765,247,992 bp assembly. The distribution of scaffold lengths is shown in dark grey with the plot radius scaled to the longest scaffold present in the assembly (100,833,644 bp, shown in red). Orange and pale-orange arcs show the N50 and N90 scaffold lengths (43,950,887 and 34,304,479 bp), respectively. The pale grey spiral shows the cumulative scaffold count on a log scale with white scale lines showing successive orders of magnitude. The blue and pale-blue area around the outside of the plot shows the distribution of GC, AT and N percentages in the same bins as the inner plot. A summary of complete, fragmented, duplicated and missing BUSCO genes in the metazoa_odb10 set is shown in the top right. An interactive version of this figure is available at https://blobtoolkit.genomehubs.org/view/wtTerLapi1_1/dataset/wtTerLapi1_1/snail.

**Figure 3.**
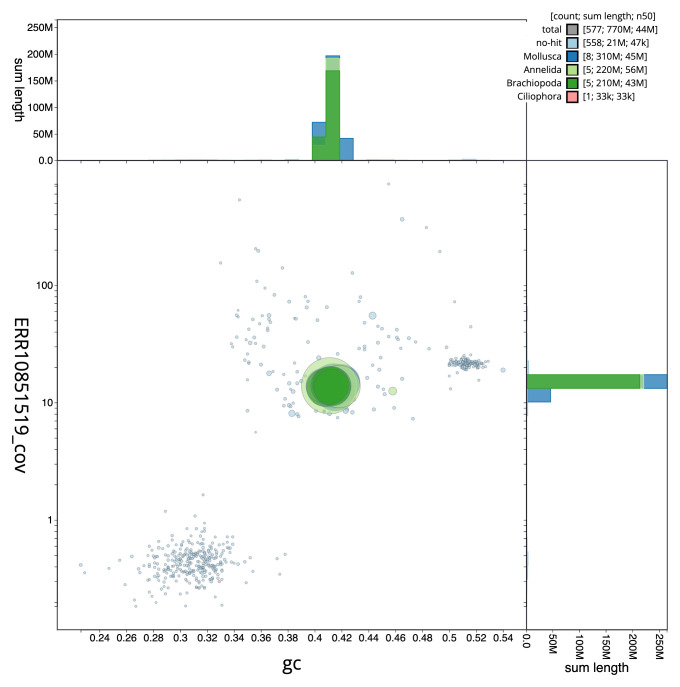
Genome assembly of *Terebella lapidaria*, wtTerLapi1.1: BlobToolKit GC-coverage plot. Sequences are coloured by phylum. Circles are sized in proportion to sequence length. Histograms show the distribution of sequence length sum along each axis. An interactive version of this figure is available at https://blobtoolkit.genomehubs.org/view/wtTerLapi1_1/dataset/wtTerLapi1_1/blob.

**Figure 4.**
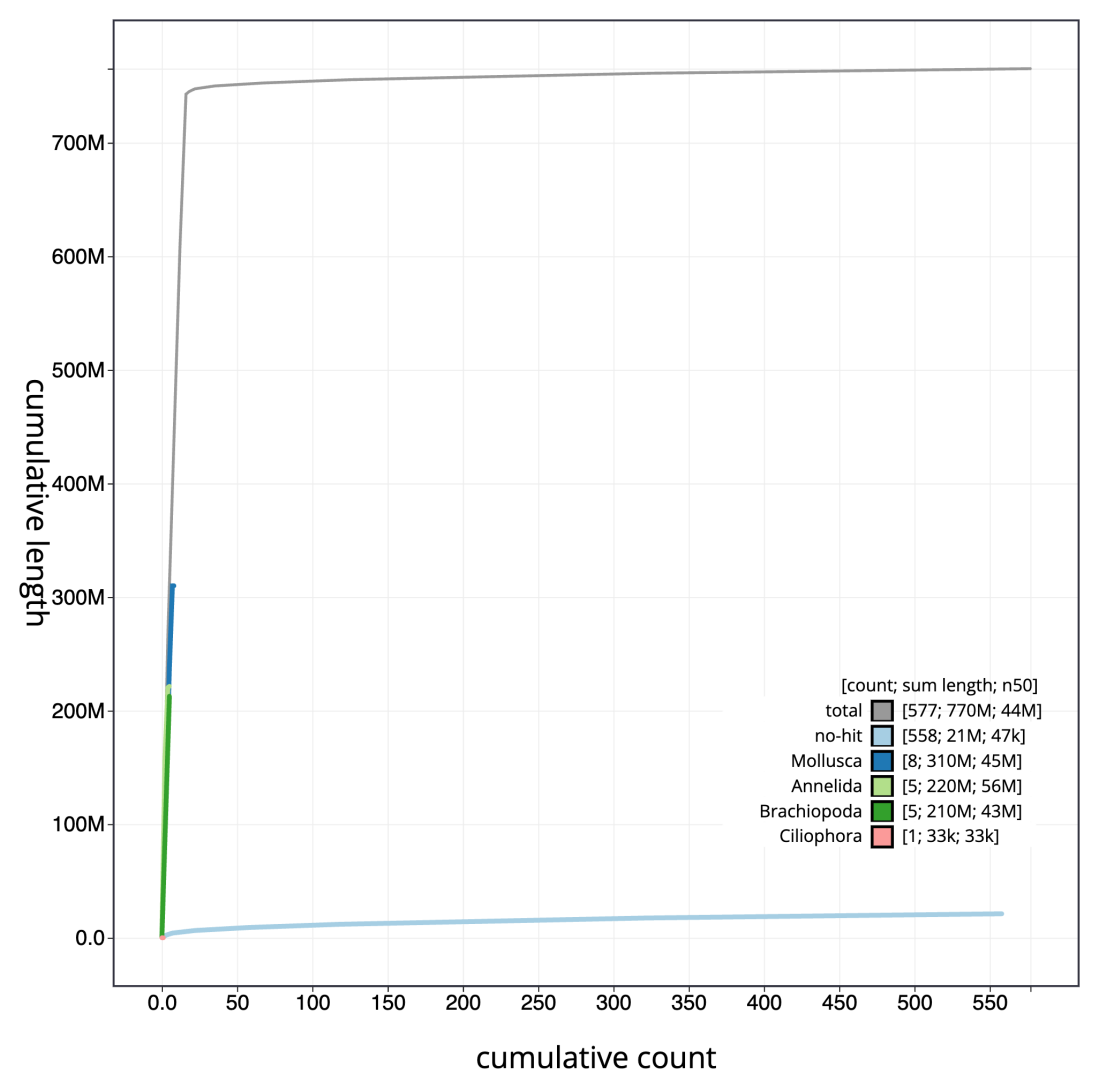
Genome assembly of *Terebella lapidaria*, wtTerLapi1.1: BlobToolKit cumulative sequence plot. The grey line shows cumulative length for all sequences. Coloured lines show cumulative lengths of sequences assigned to each phylum using the buscogenes taxrule. An interactive version of this figure is available at https://blobtoolkit.genomehubs.org/view/wtTerLapi1_1/dataset/wtTerLapi1_1/cumulative.

**Figure 5.**
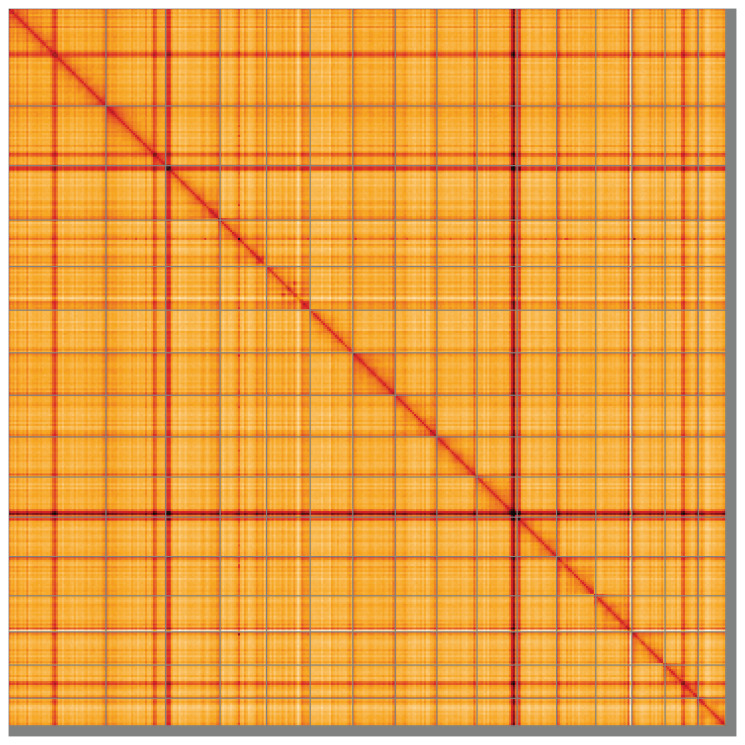
Genome assembly of *Terebella lapidaria*, wtTerLapi1.1: Hi-C contact map of the wtTerLapi1.1 assembly, visualised using HiGlass. Chromosomes are shown in order of size from left to right and top to bottom. An interactive version of this figure may be viewed at https://genome-note-higlass.tol.sanger.ac.uk/l/?d=BaRPjPiNRWWAmO2tmlp1Kw.

Manual assembly curation corrected 32 missing joins or mis-joins and 13 haplotypic duplications, reducing the assembly length by 0.8% and the scaffold number by 3.03%. The final assembly has a total length of 765.20 Mb in 576 sequence scaffolds with a scaffold N50 of 44.0 Mb ( Table 2). The snail plot in Figure 2 provides a summary of the assembly statistics, while the distribution of assembly scaffolds on GC proportion and coverage is shown in Figure 3. The cumulative assembly plot in Figure 4 shows curves for subsets of scaffolds assigned to different phyla. Most (97.22%) of the assembly sequence was assigned to 16 chromosomal-level scaffolds. Chromosome-scale scaffolds confirmed by the Hi-C data are named in order of size ( Figure 5; Table 3). While not fully phased, the assembly deposited is of one haplotype. Contigs corresponding to the second haplotype have also been deposited. The mitochondrial genome was also assembled and can be found as a contig within the multifasta file of the genome submission.

**Table 2.** Genome assembly data for *Terebella lapidaria*, wtTerLapi1.1.

Genome assembly		
Assembly name	wtTerLapi1.1	
Assembly accession	GCA_949152475.1	
*Accession of alternate haplotype*	*GCA_949152485.1*	
Span (Mb)	765.20	
Number of contigs	820	
Contig N50 length (Mb)	6.7	
Number of scaffolds	576	
Scaffold N50 length (Mb)	44.0	
Longest scaffold (Mb)	100.83	
Assembly metrics *		*Benchmark*
Consensus quality (QV)	60.0	*≥ 50*
*k*-mer completeness	100.0%	*≥ 95%*
BUSCO **	C:96.3%[S:95.0%,D:1.3%], F:1.9%,M:1.8%,n:954	*C ≥ 95%*
Percentage of assembly mapped to chromosomes	97.22%	*≥ 95%*
Sex chromosomes	None	*localised homologous pairs*
Organelles	Mitochondrial genome: 15.97 kb	*complete single alleles*

* Assembly metric benchmarks are adapted from column VGP-2020 of “Table 1: Proposed standards and metrics for defining genome assembly quality” from Rhie *et al.* (2021). ** BUSCO scores based on the metazoa_odb10 BUSCO set using version 5.4.3. C = complete [S = single copy, D = duplicated], F = fragmented, M = missing, n = number of orthologues in comparison. A full set of BUSCO scores is available at https://blobtoolkit.genomehubs.org/view/wtTerLapi1_1/dataset/wtTerLapi1_1/busco.

**Table 3.** Chromosomal pseudomolecules in the genome assembly of *Terebella lapidaria*, wtTerLapi1.

INSDC accession	Name	Length (Mb)	GC%
OX424555.1	1	100.83	41.0
OX424556.1	2	61.85	41.5
OX424557.1	3	56.41	42.0
OX424558.1	4	48.06	41.0
OX424559.1	5	45.2	41.5
OX424560.1	6	44.13	41.0
OX424561.1	7	43.95	41.5
OX424562.1	8	43.11	41.0
OX424563.1	9	41.54	42.0
OX424564.1	10	41.56	41.0
OX424565.1	11	40.88	41.0
OX424566.1	12	40.61	41.5
OX424567.1	13	37.36	41.0
OX424568.1	14	34.35	41.0
OX424569.1	15	34.3	40.5
OX424570.1	16	28.56	40.5
OX424571.1	MT	0.02	34.5

The estimated Quality Value (QV) of the final assembly is 60.0 with *k*-mer completeness of 100.0%, and the assembly has a BUSCO v5.4.3 completeness of 96.3% (single = 95.0%, duplicated = 1.3%), using the metazoa_odb10 reference set ( *n* = 954).

Metadata for specimens, BOLD barcode results, spectra estimates, sequencing runs, contaminants and pre-curation assembly statistics are given at https://links.tol.sanger.ac.uk/species/1131437.

## Methods

### Sample acquisition

Adult specimens of *Terebella lapidaria* were collected Batten Bay, Devon, UK (latitude 50.36, longitude –4.13) on 2020-11-15. The specimens were collected by Patrick Adkins, John Bishop, Nova Mieszkowska (all Marine Biological Association) and Teresa Darbyshire and Anna Holmes (both Amgueddfa Cymru) and identified by Teresa Darbyshire. The specimens were preserved by liquid nitrogen. One specimen (specimen ID MBA-201115-002C, ToLID wtTerLapi1) was used for PacBio DNA sequencing, and another (specimen ID MBA-201115-002E, ToLID wtTerLapi3) for Hi-C and RNA sequencing.

The initial species identification was verified by an additional DNA barcoding process according to the framework developed by Twyford *et al*. (2024). A small sample was dissected from the specimen and stored in ethanol, while the remaining parts of the specimen were shipped on dry ice to the Wellcome Sanger Institute (WSI). The tissue was lysed, the COI marker region was amplified by PCR, and amplicons were sequenced and compared to the BOLD database, confirming the species identification ( Crowley *et al.*, 2023). Following whole genome sequence generation, the relevant DNA barcode region was also used alongside the initial barcoding data for sample tracking at the WSI ( Twyford *et al.*, 2024). The standard operating procedures for Darwin Tree of Life barcoding have been deposited on protocols.io ( Beasley *et al.*, 2023).

### Nucleic acid extraction

The workflow for high molecular weight (HMW) DNA extraction at the WSI Tree of Life Core Laboratory includes a sequence of core procedures: sample preparation; sample homogenisation, DNA extraction, fragmentation, and clean-up. In sample preparation, the wtTerLapi1 sample was weighed and dissected on dry ice ( Jay *et al.*, 2023). Tissue from the anterior body was homogenised using a PowerMasher II tissue disruptor ( Denton *et al.*, 2023a).

HMW DNA was extracted using the Automated MagAttract v1 protocol ( Sheerin *et al.*, 2023). DNA was sheared into an average fragment size of 12–20 kb in a Megaruptor 3 system ( Todorovic *et al.*, 2023). Sheared DNA was purified by solid-phase reversible immobilisation ( Strickland *et al.*, 2023): in brief, the method employs AMPure PB beads to eliminate shorter fragments and concentrate the DNA. The concentration of the sheared and purified DNA was assessed using a Nanodrop spectrophotometer and Qubit Fluorometer using the Qubit dsDNA High Sensitivity Assay kit. Fragment size distribution was evaluated by running the sample on the FemtoPulse system.

RNA was extracted from posterior body tissue of wtTerLapi3 in the Tree of Life Laboratory at the WSI using the RNA Extraction: Automated MagMax™ *mir*Vana protocol ( do Amaral *et al.*, 2023). The RNA concentration was assessed using a Nanodrop spectrophotometer and a Qubit Fluorometer using the Qubit RNA Broad-Range Assay kit. Analysis of the integrity of the RNA was done using the Agilent RNA 6000 Pico Kit and Eukaryotic Total RNA assay.

Protocols developed by the WSI Tree of Life laboratory are publicly available on protocols.io ( Denton *et al.*, 2023b).

### Sequencing

Pacific Biosciences HiFi circular consensus DNA sequencing libraries were constructed according to the manufacturers’ instructions. Poly(A) RNA-Seq libraries were constructed using the NEB Ultra II RNA Library Prep kit. DNA and RNA sequencing was performed by the Scientific Operations core at the WSI on Pacific Biosciences Sequel IIe (HiFi) and Illumina NovaSeq X (RNA-Seq) instruments. Hi-C data were also generated from mid-body tissue of wtTerLapi3 using the Arima-HiC v2 kit. The Hi-C sequencing was performed using paired-end sequencing with a read length of 150 bp on the Illumina NovaSeq 6000 instrument.

### Genome assembly, curation and evaluation

***Assembly***

Original assembly of HiFi reads was performed using Hifiasm ( Cheng *et al.*, 2021) with the --primary option. Haplotypic duplications were identified and removed with purge_dups ( Guan *et al.*, 2020). Hi-C reads were further mapped with bwa-mem2 ( Vasimuddin *et al.*, 2019) to the primary contigs, which were further scaffolded using the provided Hi-C data ( Rao *et al.*, 2014) in YaHS ( Zhou *et al.*, 2023) using the --break option. Scaffolded assemblies were evaluated using Gfastats ( Formenti *et al.*, 2022), BUSCO ( Manni *et al.*, 2021) and MERQURY.FK ( Rhie *et al.*, 2020).

The mitochondrial genome was assembled using MitoHiFi ( Uliano-Silva *et al.*, 2023), which runs MitoFinder ( Allio *et al.*, 2020) and uses these annotations to select the final mitochondrial contig and to ensure the general quality of the sequence.

***Assembly curation***

The assembly was decontaminated using the Assembly Screen for Cobionts and Contaminants (ASCC) pipeline (article in preparation). Manual curation was primarily conducted using PretextView ( Harry, 2022), with additional insights provided by JBrowse2 ( Diesh *et al.*, 2023) and HiGlass ( Kerpedjiev *et al.*, 2018). Scaffolds were visually inspected and corrected as described by Howe *et al*. (2021). Any identified contamination, missed joins, and mis-joins were corrected, and duplicate sequences were tagged and removed. The entire process is documented at https://gitlab.com/wtsi-grit/rapid-curation (article in preparation).

***Evaluation of the final assembly***

A Hi-C map for the final assembly was produced using bwa-mem2 ( Vasimuddin *et al.*, 2019) in the Cooler file format ( Abdennur & Mirny, 2020). To assess the assembly metrics, the *k*-mer completeness and QV consensus quality values were calculated in Merqury ( Rhie *et al.*, 2020). This work was done using Nextflow ( Di Tommaso *et al.*, 2017) DSL2 pipelines “sanger-tol/readmapping” ( Surana *et al.*, 2023a) and “sanger-tol/genomenote” ( Surana *et al.*, 2023b). The genome was analysed within the BlobToolKit environment ( Challis *et al.*, 2020) and BUSCO scores ( Manni *et al.*, 2021; Simão *et al.*, 2015) were calculated.

The genome assembly and evaluation pipelines were developed using the nf-core tooling ( Ewels *et al.*, 2020), use MultiQC ( Ewels *et al.*, 2016), and make extensive use of the Conda package manager, the Bioconda initiative ( Grüning *et al.*, 2018), the Biocontainers infrastructure ( da Veiga Leprevost *et al.*, 2017), and the Docker ( Merkel, 2014) and Singularity ( Kurtzer *et al.*, 2017) containerisation solutions.

Table 4 contains a list of relevant software tool versions and sources.

**Table 4.** Software tools: versions and sources.

Software tool	Version	Source
BlobToolKit	4.2.1	https://github.com/blobtoolkit/blobtoolkit
BUSCO	5.3.2	https://gitlab.com/ezlab/busco
Hifiasm	0.16.1-r375	https://github.com/chhylp123/hifiasm
HiGlass	1.11.6	https://github.com/higlass/higlass
Merqury	MerquryFK	https://github.com/thegenemyers/MERQURY.FK
MitoHiFi	2	https://github.com/marcelauliano/MitoHiFi
PretextView	0.2	https://github.com/wtsi-hpag/PretextView
purge_dups	1.2.3	https://github.com/dfguan/purge_dups
sanger-tol/genomenote	v1.0	https://github.com/sanger-tol/genomenote
sanger-tol/readmapping	1.1.0	https://github.com/sanger-tol/readmapping/tree/1.1.0
YaHS	1.2a	https://github.com/c-zhou/yahs

### Wellcome Sanger Institute – Legal and Governance

The materials that have contributed to this genome note have been supplied by a Darwin Tree of Life Partner. The submission of materials by a Darwin Tree of Life Partner is subject to the **‘Darwin Tree of Life Project Sampling Code of Practice’**, which can be found in full on the Darwin Tree of Life website here. By agreeing with and signing up to the Sampling Code of Practice, the Darwin Tree of Life Partner agrees they will meet the legal and ethical requirements and standards set out within this document in respect of all samples acquired for, and supplied to, the Darwin Tree of Life Project.

Further, the Wellcome Sanger Institute employs a process whereby due diligence is carried out proportionate to the nature of the materials themselves, and the circumstances under which they have been/are to be collected and provided for use. The purpose of this is to address and mitigate any potential legal and/or ethical implications of receipt and use of the materials as part of the research project, and to ensure that in doing so we align with best practice wherever possible. The overarching areas of consideration are:

•     Ethical review of provenance and sourcing of the material

•     Legality of collection, transfer and use (national and international) 

Each transfer of samples is further undertaken according to a Research Collaboration Agreement or Material Transfer Agreement entered into by the Darwin Tree of Life Partner, Genome Research Limited (operating as the Wellcome Sanger Institute), and in some circumstances other Darwin Tree of Life collaborators.

## Data Availability

European Nucleotide Archive: *Terebella lapidaria* (a segmented worm). Accession number PRJEB59382; https://identifiers.org/ena.embl/PRJEB59382 ( Wellcome Sanger Institute, 2023). The genome sequence is released openly for reuse. The *Terebella lapidaria* genome sequencing initiative is part of the Darwin Tree of Life (DToL) project. All raw sequence data and the assembly have been deposited in INSDC databases. The genome will be annotated using available RNA-Seq data and presented through the Ensembl pipeline at the European Bioinformatics Institute. Raw data and assembly accession identifiers are reported in Table 1 and Table 2.

## Author information

Members of the Marine Biological Association Genome Acquisition Lab are listed here: https://doi.org/10.5281/zenodo.8382513.

Members of the Darwin Tree of Life Barcoding collective are listed here: https://doi.org/10.5281/zenodo.4893703.

Members of the Wellcome Sanger Institute Tree of Life Management, Samples and Laboratory team are listed here: https://doi.org/10.5281/zenodo.10066175.

Members of Wellcome Sanger Institute Scientific Operations: Sequencing Operations are listed here: https://doi.org/10.5281/zenodo.10043364.

Members of the Wellcome Sanger Institute Tree of Life Core Informatics team are listed here: https://doi.org/10.5281/zenodo.10066637.

Members of the Tree of Life Core Informatics collective are listed here: https://doi.org/10.5281/zenodo.5013541.

Members of the Darwin Tree of Life Consortium are listed here: https://doi.org/10.5281/zenodo.4783558.
