# Supplementary material for: The genome sequence of a segmented worm, Terebella lapidaria Linnaeus, 1767
Source: Wellcome Open Res. 2024 Aug 7;9:432. [Version 1] doi: 10.12688/wellcomeopenres.22823.1 (PMC11364976; doi:10.12688/wellcomeopenres.22823.1)
Supplement: Peer review history

# Reviewer report: Reviewer response for version 1

**DOI:** 10.21956/wellcomeopenres.25130.r95053

**Published:** 2024-09-06

**Authors:** James Wasmuth

**Affiliations:** 1 Faculty of Veterinary Medicine, University of Calgary, Calgary, Alberta, Canada

The authors present a high quality genome assembly for *Terebella lapidaria*.

**Background**

 The presented rationale for creating a genome assembly for *T. lapidaria *could be improved. The authors should elaborate on "[T]he first of its kind for this species." At which taxonomic rank is this assembly the first? Are there additional compelling reasons for sequencing this species: conservation, invasive species, model of animal development/biochemistry? I'm genuinely interested.

**Data report**

 The authors should comment on the high proportion of *contamination* from Brachiopoda that is identified by Blobtools (Figure 3). Further, in Figure 4 the light green Annelida line obscured by the darker green Brachiopod line.

**Methods**

 BUSCO: The authors need to confirm which version of they used: in the main text 5.4.3 vs in table 4 5.3.2. They should also specify which alignment tools they used within BUSCO (e.g. augustus vs metaeuk) as these can lead to considerable differences in the score.

**Other considerations**

 Given that the authors carried out RNA-Seq, I would have welcomed a first pass at genome annotation; how many protein coding genes could be prediction.

 The mitochondrial genome has been assembled but no annotation is presented, e.g. size, tRNAs, genes.

Are sufficient details of methods and materials provided to allow replication by others?

Partly

Is the rationale for creating the dataset(s) clearly described?

Partly

Are the datasets clearly presented in a useable and accessible format?

Yes

Are the protocols appropriate and is the work technically sound?

Yes

Reviewer Expertise:

Invertebrate genomics, transcriptomics, evolutionary biochemistry

I confirm that I have read this submission and believe that I have an appropriate level of expertise to confirm that it is of an acceptable scientific standard.

# Reviewer report: Reviewer response for version 1

**DOI:** 10.21956/wellcomeopenres.25130.r93506

**Published:** 2024-08-30

**Authors:** Mehmet Dayi

**Affiliations:** 1 Düzce University, Düzce,, Turkey

**Background section**

 For the figure 1 (specimen figure) it would be good to use a bar describing length of the specimen since the authors mention about different lenght of this species *(....They can reach 80–160 segments in size and up to 9 centimetres in length (Fauvel, 1927)....).*

**Genome sequence report**

 The BUSCO tool version number should be checked and be consistent with that mentioned in Table 4. 

**Assembly curation section**

 The authors should provide more details about decontamination of the assembly since they used their own work which is in preparation (article in preparation). .... *The assembly was decontaminated using the Assembly Screen for Cobionts and Contaminants (ASCC) pipeline (article in preparation)....* 

 Additionally, the authors provide more details and clear explanation of the entire process. In the current form of the method it is not clear since the authors again used their unpublished work (article in preparation). Besides, the link (https://gitlab.com/wtsi-grit/rapid-curation) provided for the entire process does not clearly show the entire process.

Are sufficient details of methods and materials provided to allow replication by others?

Partly

Is the rationale for creating the dataset(s) clearly described?

Partly

Are the datasets clearly presented in a useable and accessible format?

Yes

Are the protocols appropriate and is the work technically sound?

Yes

Reviewer Expertise:

Genome evolution.

I confirm that I have read this submission and believe that I have an appropriate level of expertise to confirm that it is of an acceptable scientific standard.

# Reviewer report: Reviewer response for version 1

**DOI:** 10.21956/wellcomeopenres.25130.r95060

**Published:** 2024-08-29

**Authors:** Maria Nilsson

**Affiliations:** 1 Senckenberg Biodiversity and Climate Research Centre, Frankfurt am Main, Germany

The nuclear genome has been sequenced from the marine segmented worm Terebella lapidaria. The species occur in the mediterranean but is also found in French and UK waters. Two individuals were used for the genome assembly. The individuals were DNA barcoded before the nuclear genome was sequenced.

 The assembly is based on pacbio longread sequences and HiC. The final length of the manual curated assembly is 765Mb and contain 16 chromosomal pseudomolecules. It was not possible to assemble the sex chromosomes. The contig N50 length is 6.7 Mb. The BUSCO score, which counts the number of complete benchmarking genes is 96.3%.

 The overall QV value is 60. The statistics indicate that it is a good assembly which is generally difficult to achieve from marine soft-bodied animals.

**Minor comments:**

 1) in the background it is stated that the genome assembly of Terebella lapidaria is "the first of its kind". Please clarify in what sense it is the first, is it the first from that family, genus etc.?

Are sufficient details of methods and materials provided to allow replication by others?

Yes

Is the rationale for creating the dataset(s) clearly described?

Yes

Are the datasets clearly presented in a useable and accessible format?

Yes

Are the protocols appropriate and is the work technically sound?

Yes

Reviewer Expertise:

phylogenomics, transposable elements, mitogenomics

I confirm that I have read this submission and believe that I have an appropriate level of expertise to confirm that it is of an acceptable scientific standard.
